# Emergence of serogroup C meningococcal disease associated with a high mortality rate in Hefei, China

**DOI:** 10.1186/1471-2334-12-205

**Published:** 2012-09-04

**Authors:** Xi-Hai Xu, Ying Ye, Li-Fen Hu, Yu-Hui Jin, Qin-Qin Jiang, Jia-Bin Li

**Affiliations:** 1 Department of Infectious Diseases, The First Affiliated Hospital of Anhui Medical University, Hefei, Anhui, China; 2 Anhui Center for Surveillance of Bacterial Resistance, Hefei, Anhui, China; 3 Department of Center Laboratory, The First Affiliated Hospital of Anhui Medical University, Hefei, Anhui, China; 4 Anhui Provincial Center for Disease Control and Prevention, Hefei, Anhui, China

**Keywords:** Neisseria meningitidis, Serogroup C strain, Incidence

## Abstract

**Background:**

*Neisseria meningitidis* serogroup C has emerged as a cause of epidemic disease in Hefei. The establishment of serogroup C as the predominant cause of endemic disease has not been described.

**Methods:**

We conducted national laboratory-based surveillance for invasive meningococcal disease during 2000–2010. Isolates were characterized by pulsed-field gel electrophoresis and multilocus sequence typing.

**Results:**

A total of 845 cases of invasive meningococcal disease were reported. The incidence increased from 1.25 cases per 100,000 population in 2000 to 3.14 cases per 100,000 in 2003 (p < 0.001), and peaked at 8.43 cases per 100,000 in 2005. The increase was mainly the result of an increase in the incidence of serogroup C disease. Serogroup C disease increased from 2/23 (9%) meningococcal cases and 0.11 cases per 100,000 in 2000 to 33/58 (57%) cases and 1.76 cases per 100,000 in 2003 (p < 0.01). Patients infected with serogroup C had serious complications more frequently than those infected with other serogroups. Specifically, 161/493 (32.7%) cases infected with serogroup C had at least one complication. The case-fatality rate of serogroup C meningitis was 11.4%, significantly higher than for serogroup A meningitis (5.3%, p = 0.021). Among patients with meningococcal disease, factors associated with death in univariate analysis were age of 15–24 years, infection with serogroup C, and meningococcemia.

**Conclusions:**

The incidence of meningococcal disease has substantially increased and serogroup C has become endemic in Hefei. The serogroup C strain has caused more severe disease than the previously predominant serogroup A strain.

## Background

Meningococcal disease is an important cause of meningitis and sepsis in children and young adults in China and worldwide. In China, this disease previously occurred in a cyclical pattern at intervals of 8–10 years [1]. Nationwide epidemics occurred in 1959, 1967, 1977, and 1984 [2,3]. Since a national immunisation campaign with meningococcal A polysaccharide vaccine was initiated in 1982, no countrywide epidemic has occurred, and morbidity rates of meningococcal disease in China have remained relatively stable at 0.2–1 cases per 100,000 during the past two decades [3-5]. During this time, serogroup A meningococci was most common serogroup to cause disease and was responsible for more than 95% of cases, whereas serogroups B cause only sporadic cases and serogroup C was even rarer [4,5]. However, since 2003 the number of meningococcal meningitis cases attributable to serogroup C has increased substantially in China [6]. The sudden increase in the number of cases due to serogroup C meningococci first occurred in China’s Anhui Province during 2003–2004. Hefei, the capital of Anhui Province—a region situated in the middle China with a population of 1.85 million in 2006—had one of the first Chinese outbreaks of meningococcal disease caused by serogroup C [6]. The incidence of meningococcal disease due to serogroup C increased rapidly, and *Neisseria meningitidis* serogroup C emerged as a new epidemic serogroup in Hefei and other areas of Anhui Province [7]. To control this disease, mass vaccination using polysaccharide vaccine for serogroup A plus C in students and children younger than 6 years was undertaken starting in 2004 in Anhui Province [7] Chemoprophylaxis was given to close contacts of patients, and a nationwide surveillance programme was implemented [7]. No epidemics of meningococcal disease caused by serogroup C occurred in China before 2002, and few reports on its epidemiological characteristics in China have been published [5]. In this article, we report the replacement of serogroup A disease by serogroup C in Hefei, China. This led to an increased incidence of disease among young infants and higher case fatality rates in the general population.

## Methods

### Case reporting and laboratory procedures

The protocol of the study adhered to the tenets of the Declaration of Helsinki and was approved by the ethics committee of Anhui Medical University. In the National Notifiable Disease Surveillance System in Hefei, patients with sudden onset of fever, headache, nausea, vomiting, stiff neck, petechial rash, accompanied by delirium, coma or shock, or Gram negative diplococci found in a smear of cerebrospinal fluid (CSF) by Gram stain should be reported within six hours to the Center for Disease Control (CDC), Hefei as suspected cases of invasive meningococcal disease. In addition to routine examination and culture in each hospital, blood and/or CSF from the suspected *N. meningitidis* patient was sent at room temperature to the bacteriology laboratory of Hefei CDC as soon as possible for bacterial culture and serogroup classification. Within 12 hours of the suspected case being reported, staff at the local Health Bureau conducted a case investigation to understand the detailed travel history and identify every close contact during the two weeks before disease onset. If *N. meningitidis* was isolated and the patient had compatible clinical symptoms and signs, the patient was a confirmed case of invasive meningococcal disease. The identification of all isolates were confirmed at Hefei CDC using conventional biochemical methods [8]. An agglutination test (Murex Biotech Ltd, Dartford, UK) for standard serogroup testing was also performed at Hefei CDC. Serogroups were determined by slide agglutination with polyclonal antibodies to capsular polysaccharides A, C, X, Y, Z, and W135 and monoclonal antibodies to polysaccharide B.

The minimal inhibitory concentrations (MICs) of the antimicrobial agents were determined for each isolate by Mueller-Hinton agar (OXOID Company, Cambridge, UK) macrodilution method described by the CLSI (formerly the National Committee for Clinical Laboratory Standards) [9]. The MIC was defined as the lowest concentration of drug that resulted in no visible growth after 18 h of incubation at 35°C in ambient air. Quality control strains were included with every batch of clinical isolates to ensure accuracy and comparable performance of the assays.

### Definitions

Cases were defined as those from which *N. meningitidis* was isolated from normally sterile specimens (e.g., CSF, blood, or joint fluid specimens). In addition, in 2004 the definition for case patients was broadened to include patients with culture-negative specimens that yielded positive results by latex agglutination and Gram stain microscopy or by latex agglutination and polymerase chain reaction. Bacteria were identified according to standardized procedures [8]. Laboratory-confirmed meningococcal meningitis was defined as growth of *N. meningitidis* on CSF cultures (with or without growth on blood cultures). Laboratory-confirmed meningococcemia was defined as growth of *N. meningitidis* on blood cultures (without growth on CSF cultures).

### Clinical information collection

We reviewed the available medical information of all cases reported to Hefei CDC from 1 January, 2000 to 30 December, 2010. The captured data included patients' history, clinical manifestations and complications, laboratory results, therapy, and outcome. Patients were categorized as either meningitis or meningococcemia.

### Incidence

We calculated the incidence based on the number of laboratory-confirmed cases reported each year from 1 January through 31 December divided by midyear population estimates for each year, as supplied by Statistics Hefei. In 2006, the estimated population of Hefei was 1.85 million.

### Statistical analysis

Serogroup C disease was compared with serogroup A disease alone, because serogroup A has specific epidemiological features and had previously been predominant in Hefei. The prevalence of other serogroups in the area did not vary over time and caused minimal disease. Univariate assessments of characteristics associated with disease due to serogroup C infection and disease resulting in death were performed using Fisher’s exact test or the Mantel-Haenszel test for categorical variables. Variables available for evaluation as potential risk factors included age group, sex, and syndrome (laboratory confirmed meningitis versus meningococcemia). Multivariable logistic regression models were fitted to the data, starting with all variables that were significant at p < 0.05 on univariate analysis and dropping non-significant factors with stepwise backward selection. Independence of data was assumed, because the vast majority of cases were considered to be unrelated. The criterion of significance was p < 0.05 in a two-tailed test. Statistical analyses were performed using SPSS V11.0 software (SPSS Inc, 2000).

## Results

### Incidence

**Figure 1:**
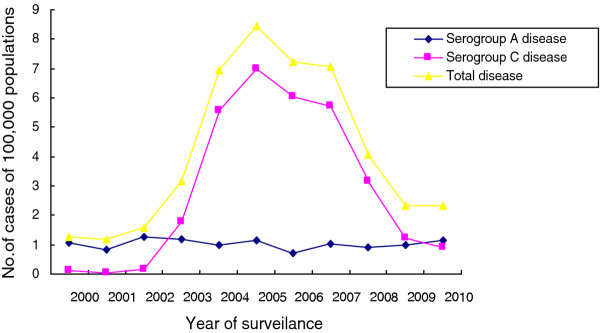
Incidence of laboratory-confirmed invasive meningococcal disease by serogroup in Hefei, China, as reported to the Hefei CDC for Communicable Diseases.

During 2000–2010, a total of 845 cases of meningococcal disease were reported to Hefei CDC. Table 1 shows the incidence of meningococcal disease over this time period. Rates of reported disease increased from 1.25 cases per 100,000 in 2000 to 3.14 cases per 100,000 in 2003 (p < 0.01). In 2005, the incidence peaked at 8.43 cases per 100,000. The increase was mainly the result of an increase in the incidence of serogroup C disease (Figure 1). The percentage of cases of serogroup C increased from 2/23 (9%) of cases and 0.11 cases per 100,000 in 2000 to 33/58 (57%) of cases and 1.76 cases per 100,000 in 2003 (p < 0.01). During 2004–2007, the incidence of serogroup C exceeded 5.50 per 100,000, whereas the incidence of other serogroups remained relatively stable (p > 0.05). The incidence of serogroup C steadily decreased in the later part of the study period, from 3.14 cases per 100,000 in 2008 to 0.92 cases per 100,000 in 2010 (p < 0.01). During the study period, the incidence of serogroup A disease remained stable and the proportion of serogroup A cases fluctuated markedly by year. For the period 2000–2002, a total of 59/74 (80%) cases were due to serogroup A, with the number decreasing to 22/58 (38%) cases in 2003. During the period 2004–2007, the percentage of serogroup A was 10%–15%.

**Table 1:** Number and incidence of laboratory-confirmed invasive meningococcal cases in Hefei reported to The Hefei CDC, by serogroup, 2000–2010

**Year of surverillance**												
**Serogroup**	**2000**	**2001**	**2002**	**2003**	**2004**	**2005**	**2006**	**2007**	**2008**	**2009**	**2010**	**Total**
A	20(87)	15(68)	24(83)	22(38)	18(14)	21(13)	13(10)	19(15)	17(22)	18(41)	21(49)	208
C	2(9)	1(5)	3(10)	33(57)	103(80)	129(83)	112(84)	106(81)	55(71)	23(52)	17(40)	584
B	1(4)	3(14)	2(7)	1(2)	a	4(3)	3(2)	5(4)	2(3)	a	3(7)	24
X	a	3(14)	a	2(3)	4(3)	2(1)	3(2)	a	1(1)	2(6)	1(2)	18
Y	a	a	a	a	3(2)	3(2)	1(1)	1(0.8)	2(3)	1(2)	a	11
Total	23	22	29	58	128	156	134	131	77	44	43	845
Incidence	1.25	119	1.57	3.14	6.92	8.43	7.24	7.08	4.06	2.34	2.32	NA

**NOTE.** Data are no. (%) of cases, unless otherwise indicated.

NA, not applicable. a,no isolates identified.

Of the meningococcal strains that were isolated during this study we analyzed 374 for drug resistance. Sixty-four (17%) meningococcal isolates were not susceptible to penicillin. The proportion of resistant isolates fluctuated by year, from 2/17 (11.8%) isolates in 2000 to a peak of 16/78 (20.5%) isolates in 2005. Penicillin-resistant isolates of all serogroups were found. Nine of 97 (9.3%) serogroup A isolates and 51/264 (19.3%) serogroup C strains (p = 0.023) were not susceptible to penicillin. The rate of trimeth-sulfa resistant strains was below 25% in serogroup A cases and above 50% in serogroup C cases. All 374 isolates were susceptible to ceftriaxone, cefotaxime and cefoxitin, and only 10 isolates were resistant to rifampin.

### Age, sex, and seasonal distribution

**Figure 2:**
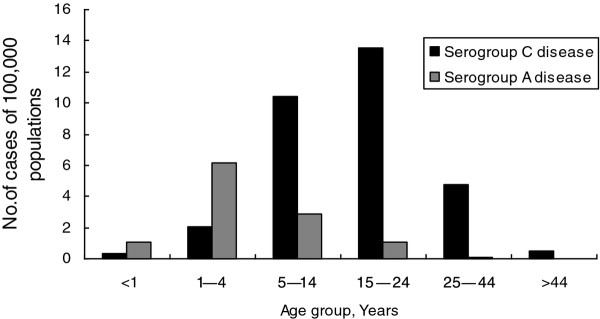
Annual age-specific incidence of confirmed serogroup C and A invasive meningococcal disease in Hefei, China, 2000-2010.

In children younger than 14 years, thirty-one percent (245/792) of cases were serogroup C, and serogroup A primarily occurred in this age group (Figure 2). Serogroup C and serogroup A demonstrated different distributions across age groups. The median age of patients with serogroup C disease was 19 years (interquartile range, 6 months–78 years), compared with 2 years (interquartile range, 2 months–31 years) for serogroup A disease (p < 0.05). A higher proportion of serogroup C disease occurred in relatively older age groups. The highest incidence (13.57 cases per 100,000) and highest proportion (43.0%) for serogroup C were the 15–24-year age group, compared with the highest incidence (6.16 cases per 100,000) and highest proportion (54.8%) for serogroup A in the 1–4-year age group (Figure 2). A total of 443 cases (75.9%) of serogroup C diseases occurred among persons aged 5–24 years, whereas the number was only 73 cases (35.1%) for serogroup A diseases. The age of serogroup A cases was younger and 186/208 (89.4%) cases of serogroup A disease occurred in children younger than 14 years, compared with 236/ (40.4%) cases for serogroup C diseases (p < 0.05). The incidence of serogroup C disease increased for all age group.

Of the 792 serogroup C and serogroup A cases, 444 (56.1%) were male and 348 (43.9%) were female. The distribution showed no significant difference between the two groups (Mann–Whitney U test: p = 0.33).

The monthly distribution of patients shows that the lowest incidence is in the summer months and the highest incidence is in the late winter months (data not shown). There were no statistically significant differences in seasonal characteristics between cases infected with serogroup C and serogroup A (data not shown).

### Comparison of clinical features between serogroup C and serogroup A cases

Complete detailed clinical information was available on 84% (493/584) of patients with serogroup C and 85% (177/208) of patients with serogroup A. The frequency of presenting symptoms, sign, and laboratory findings of the patients are shown in Table 2. Patients with serogroup C disease tended to have more petechiae and/or purpura (80.3% versus 73.4%; OR: 1.46; 95% CI: 0.98–2.18), but the difference did not achieve statistical significance. When compared with serogroup A disease, serogroup C disease was more likely to cause meningococcemia (76.9% versus 48.0%; OR: 3.60; 95%CI: 2.51–5.17; p < 0.001). Patients with serogroup C were more likely to have a decreased platelet count (26.7% versus 8.5%, p < 0.001). Patients infected with serogroup C were more frequently involved in serious complications; 161/493 (32.7%) cases infected with serogroup C had at least one complication, whereas only 23/177 (13.0%) of serogroup A cases had complications (p < 0.001). Patients in serogroup C were significantly more likely than those in serogroup A to have shock (18.5% versus 7.3%; p <0.001) and disseminated intravascular coagulation (12.2% versus 4.0%; p = 0.002). Of 18 cases with kidney failure or multiple organ failure, 14 (77.8%) had serogroup C disease.

**Table 2:** **Clinical manifestations, laboratory data, and outcome of 670 meningococcal disease patients with completed case records: serogroup C*versus*A**

**Feature**	**Serogroup C (n = 493)**	**Serogroup A (n = 177)**		**P**
onset				
Presentation days (mean ± SD)	1.8 ± 2.5	1.9 ± 2.4		0.810
Fever	481(97.6)	169(95.5)		0.162
Nausea and/or vomiting	141(28.5)	55(31.2)		0.535
Headache	165(33.4)	52(29.1)		0.319
Petechia and/or purpura	396(80.3)	126(73.4)		0.063
Altered mental status	136(27.6)	49(27.7)		0.980
Disease classification				
Meningitis	114(23.1)	92(52.0)		<0.001
Meningococcemia	379(76.9)	85(48.0)		
With Shock	91(18.5)	13(7.3)		<0.001
With pneumonia	12(2.8)	3(1.7)		0.784
With arthritis	9 (1.8)	4(2.3)		0.967
Complications	161	23		<0.001
Shock	91(18.5)	13(7.3)		<0.001
DIC	60(12.2)	7(4.0)		0.002
kidney failure	10(2)	3(1.7)		0.783
MOF	4(0.8)	1(0.6)		1.0
WBC count				
>10 × 10^9^/L	302(61.3)	99(56.1)		0.019
10~4 × 10^9^/L	117(23.7)	35(19.8)		
<4 × 10^9^/L	74(15.0)	43(24.3)		
Platelet count				
>100 × 10^9^/L	361(73.2)	162(91.5)		<0.001
100~50 × 10^9^/L	87(17.6)	11(6.2)		
100~50 × 10^9^/L	87(17.6)	11(6.2)		
CSF data (mean ± SD)				
Glucose level(mmol/L)	2.07 ± 1.7	1.91 ± 1.8		0.717
CSF glucose / blood glucose ratio	0.24 ± 0.23	0.21 ± 0.29		0.438
Total protein level(g/L)	3.41 ± 2.9	3.17 ± 2.18		0.106
Lactate level(mmol/L)	10.4 ± 9.2	11.0 ± 8.9		0.113
WBC count(10^9^/L)	5.3 ± 13.8	8.1 ± 13.6		0.895
Sequelae	46/484(9.5)	6/171(3.5)		0.013
Scars	19/484(3.9)	2/171(1.2)		0.132
Amputations	14/484(2.9)	0		0.026
sensorineural hearing loss	5/484(1.0)	3/171(1.8)		0.739
Others	8/484(17)	1/171(0.6)		0.516
Death	55/484(11.4)	9/171(5.3)		0.021

NOTE. Data are no. (%) of patients, unless otherwise indicated.

*WBC*: white blood cells. *DIC*: disseminated intravascular coagulation.

*MOF*: multiple organ failure.

There was adequate information to objectively assess sequelae in 655 survivors. About nine percent of serogroup C cases (46 of 484 cases) and 4% of serogroup A cases (6 of 171) were affected (p = 0.013). For survivors of serogroup C disease, skin scars (3.9%) and amputations (2.9%) were the most frequent problems observed. Other problems of serogroup C survivors were permanent minor reduction in kidney function (n = 3) permanent knee damage following septic arthritis causing mild reduction in mobility (n = 2), partial paralysis (n = 1), and speech problem (n = 1). One survivor of serogroup C disease developed multiple neural deficits following an intracerebral hemorrhage.

During the period of study, a total of 64 fatal cases were reported to Hefei CDC. During 2000–2002, only four fatal cases were reported and the case-fatality rate remained relatively low (4/74 cases; 5.4%). However, case-fatality rates increased to 15.5% (9/58) in 2003 and peaked at 17.3% (22/127 This increase was mainly due to increasing deaths caused by serogroup C disease. Table 3 shows that the case-fatality rate of serogroup C meningitis was 11.4%, significantly higher than serogroup A meningitis (5.3%, p = 0.021). Among patients with invasive serogroup C or A disease, factors associated with death in univariate analysis were age of 15–24 years, infection with serogroup C, and meningococcemia (Table 3). In multivariable analysis, age group and meningococcemia were significantly associated with death. There was a marginal association between serogroup C infection and an increased risk of dying (adjusted OR, 2.73; p *=* 0.052).

**Table 3:** Univariate and multivariable analyses of risk factors for death in serogroup C and A meningococcal infection in Hefei, 2000–2010

**Characteristic**	**Mortality rate, no. of deaths/no. of patients (%)**	**Univariate analysis**		**Multivariable analysis**	
		**OR (95% CI)**	**P**	**OR (95% CI)**	**p**
Sex					
Female	25/263(9.5)	Reference			
Female	25/263(9.5)	Reference			
Age, years					
<1	4 /23(1.7)	Reference	<0.001	Reference	<0.001
1-4	10/126(7.9)	0.08(0.03-0.13)		0.20 (0.11–0.38)	
5-14	12/209(5.7)	0.06(0.02-0.1)		0.13 (0.07–0.27)	
15-24	37 /224(16.5)	0.17(0.13-0.2)		0.97 (0.24–3.86)	15-24
25-44	1/66(1.5)	0.02(0.01-0.09)		0.13 (0.07–0.27)	25-44
>44	0/7(0)	Undefined		Undefined	>44
Syndrome ^a^					
Meningitis	9/200(4.5)	Reference	0.003	Reference	0.002
Meningococcemia	55/455(12.1)	2.92(1.41-6.02)		3.21 (0.89–11.56)	
Serogroup					
A	9/171(5.3)	Reference	0.021	Reference	0.053
C	55/484(11.4)	2.31(1.12-4.78)		2.73 (0.69–10.87)	
Strain’s susceptibility to penicillin					
Susceptible	37/288(12.8)	Reference	0.388		
Non-susceptible	9/52(17.3)	0.70(0.32-1.56)			

^a^ Two cases of arthritis with positive synovial fluid culture results were excluded.

## Discussion

Although *N. meningitidis* serogroup C is a prominent serogroup in many regions of the world and has occasionally caused epidemics and frequently causes outbreaks [10], only sporadic cases and no epidemics of meningococcal disease due to serogroup C occurred in China before 2002. In this study, we report the emergence of endemic serogroup C meningococcal disease and epidemiological characteristics in China. The rate of invasive meningococcal disease in Hefei increased 2.5 times from 2000 to 2003, and the incidence peaked at 8.43 cases per 100,000 in 2005. This increase was mostly because of the emergence and increase in the incidence of serogroup C disease.

The reasons for the emergence and increase in serogroup C disease in Anhui Province and parts of China (while other potentially epidemic strains have remained relatively quiescent) are not fully understood. It is well-known that molecular mechanisms likely play an important role in the epidemiology of meningococcal disease. *N. meningitidis* uses several mechanisms to change its characteristics, such as antigenic structure and resistance to antibiotics. Many of these changes occur through horizontal gene transfer, by which the organism obtains large DNA sequences from other meningococcal strains or other species [11]. Capsular switching is a genetic mechanism that allows *N. meningitidis* to change its capsular phenotype, permiting immunologic escape [12,13]. *N. meningitidis* outbreaks can be started or sustained when this occurs. Capsular switching appears to have been responsible for an outbreak of serogroup W-135 disease during the 2000 Hajj in Mecca, Saudi Arabia. Subsequent to this outbreak, the epidemic strain spread globally and, in one example, caused an epidemic in Burkina Faso [14,15]. Selected isolates of the serogroup C clone from Hefei and the rest of Anhui Province were found to belong to ST-4821, a unique clone of serogroup C that does not belong to any of the previously reported sequence types [16]. For this reason, capsular switching may have been a cause for the emergence of serogroup C in Anhui Province. Published data showed that this unique ST-4821 clone was first identified in Anhui Province during 2003–04, and the same ST clones were also identified in 11 other provinces in China during 2004–2005 [7,16,17].

The overall case-fatality rate in Hefei tripled from 2000 to 2003. We report that the rate of case-fatality with serogroup C was 2.2 times that of serogroup A during the period covered. This was of borderline statistical significance and factors associated with death in univariate analysis were age of 15–24 years, infection with serogroup C, and meningococcemia. The results of this study confirm the severity of serogroup C disease [18,19]. The reason for the high virulence of this particular strain of *N. meningitidis* is unknown, but the strain is associated with a high frequency of meningococcemia with severe complications, which was of statistical significance in multivariable analysis. Meningococcemia was also independently associated with serogroup C disease and with increased risk of death. Meningococcemia has a higher case-fatality rate than meningitis [20].

Few published papers discuss the clinical characteristics of serogroup C meningococcal disease [18-20]. We reviewed the detailed clinical information and found serogroup C disease more likely to cause meningococcemia, decreased platelet counts, and serious complications. Our findings provide valuable information to understand the severity of serogroup C disease. Several scoring systems have been developed for assessing the prognosis and mortality of meningococcal disease [21-24]. The Glasgow Meningococcal Septicemia Prognostic Score is a good example of a clinical prediction tool for assessing the mortality of meningococcal disease [21,22]. Most of those scoring systems are based on easily available clinical and laboratory parameters such as age distribution, period between onset of disease and admission, absence of meningitis, presence of widespread skin lesions, hypotension, metabolic acidosis, normal C-reactive protein level, absence of leukocytosis, presence of thrombocytopenia, and hypofibrinogenemia [21-24]. However, some of the above factors (e.g., C-reactive protein and fibrinogen levels) were not routinely checked in our cases.

Of note, the age distribution of patients shifted from younger to older age categories in the Hefei area during the study period. From 1989 to 1999, 78% of cases of meningococcal disease occurred in children younger than 14 years [4]. However, the proportion of cases in this age group decreased to 31% from 2000 to 2010, while the proportion in 15–24 year olds increased from 22% (1989–1999) to 52.6% (2000–2010). The elevated incidence of meningococcal disease among adolescents and young adults has also been noted in previous studies [15,25]. Some reports indicated that the age distribution was not uniform for all serogroups, and disease caused by serogroup C was more likely to affect older children than disease caused by serogroup B [26]. However, another study regarded the shift toward older age as a characteristic of a meningococcal disease epidemic, and suggested that the age distribution of the disease would return to “normal” after the epidemic [27]. Possible explanations for the change in age group affected include: *N meningitidis* of serogroup C was antigenically new to the entire population; adolescents and young adults have enhanced risk factors for meningococcal transmission and invasion, such as crowding, active or passive smoking, and exposure to oral secretions. Understanding the age distribution of meningococcal disease is necessary to devise control and preventive measures such as vaccinations for high-risk populations. Because serogroup C is a vaccine-preventable strain, our findings stress the importance of further study of the age distribution of this disease. In the early days of an outbreak of serogroup C meningococcal disease (2003–2004) in Anhui Province, serogroup C vaccine was only recommended for children younger than two years. In 2005 serogroup C meningococcal conjugate vaccine was introduced to all those who were younger than 25 years, especially for those populations such as middle school students, college freshmen and service personnel. From September 2003 to July 2007, 627,000 doses of serogroup A and C vaccine were used in Hefei, providing an estimated 37–51% coverage of the population aged 2–24 years. After the immunization campaign in Hefei, the incidence of serogroup C meningococcal disease decreased. In addition, serogroup C meningococcal pharyngeal carriage has likely been reduced, which has likely led to a reduction in serogroup C meningococcal disease in the unimmunized population.

## Conclusion

The incidence of meningococcal disease substantially increased in Hefei from 2000 through 2010. This was mainly the result of an increase in the incidence of serogroup C disease, and serogroup C has become endemic in Hefei. Patients infected with serogroup C had greater disease severity more frequently than those infected with serogroup A, the previous predominant serogroup.

## Competing interests

The author(s) declare that they have no competing interests.

## Authors’ contributions

X-XH and YY participated in the design of the study and data analysis. JYH and JQ-Q carried out the data collection. HL-F participated in the study of complements. LJ-B conceived of the study, participated in its design and coordination, and helped to draft the manuscript. All authors read and approved the final manuscript.

## Pre-publication history

The pre-publication history for this paper can be accessed here:

http://www.biomedcentral.com/1471-2334/12/205/prepub
